# Combining hypnotics in general anesthesia: pharmacokinetic and pharmacodynamic perspectives on propofol and remimazolam

**DOI:** 10.1186/s40981-026-00887-z

**Published:** 2026-10-28

**Authors:** Shinju Obara

**Affiliations:** Department of Anesthesiology, Fukushima Medical University School of Medicine, 1 Hikarigaoka, Fukushima, Fukushima 960-1295 Japan

**Keywords:** Propofol, Remimazolam, Drug interaction, Pharmacodynamics, Response surface model

Combining two hypnotics is an attractive option when increasing the dose of a single agent causes hypotension or an undesirable recovery profile. Propofol and remimazolam can both be used to maintain general anesthesia, but they differ in their hemodynamic effects and the availability of pharmacological reversal. The central question is whether combining these agents can improve the balance between hypnosis, adverse effects, and emergence by reducing the required concentration of each agent. Dose comparisons alone cannot answer this question. The effects of the combination must be considered in relation to drug concentrations, the responses being assessed, and the time course of administration.

Studies published in the *Journal of Anesthesia* and *JA Clinical Reports* provide clinical context for this question. In a multicenter trial, Doi et al. reported that remimazolam was noninferior to propofol in anesthetic efficacy and was associated with a lower incidence of hypotension but a longer time to extubation [1]. These differences provide a rationale for investigating the combined use of propofol and remimazolam.

Katsuragawa et al. retrospectively evaluated combined administration during anesthesia maintenance [2]. After propensity score matching, the remimazolam-only, propofol-only, and combination groups each included 75 patients. Median extubation times were 9, 13, and 9 min, respectively. However, flumazenil was administered to 97.3% of patients in the remimazolam-only group and 92.0% of those in the combination group. In the latter group, propofol was discontinued a median of 4 min before remimazolam. Thus, the observed differences in extubation time reflect management that included drug discontinuation and reversal. They cannot be interpreted as evidence that the combination itself accelerates emergence without pharmacological reversal. Hypotension was assessed using the time-weighted average area under the threshold (TWA-AUT), calculated by dividing the area below a mean arterial pressure of 65 mmHg by the duration of surgery. Median TWA-AUT values were 0.74, 2.03, and 1.06 mmHg in the remimazolam-only, propofol-only, and combination groups, respectively. A significant difference was found only between the two single-hypnotic groups, not between the propofol-only and combination groups [2].

Synergistic hypnotic effects of midazolam and propofol have previously been reported [3]. However, remimazolam’s classification as a benzodiazepine does not establish the magnitude or clinical relevance of its interaction with propofol. Lyu et al. studied 150 patients who received remimazolam, propofol, or their combination 90 s after intravenous sufentanil 0.1 μg/kg, using loss of the eyelash reflex as the endpoint. After determining the median effective dose (ED_50_) of each hypnotic alone, they fixed the remimazolam dose at 25%, 50%, or 75% of its ED_50_, estimated the ED_50_ of propofol in each combination, and performed an isobolographic analysis [4]. Among the combinations studied, remimazolam 0.032 mg/kg and propofol 0.221 mg/kg, a dose ratio of approximately 1:7, produced the largest interaction coefficient, indicating statistically significant synergy. This was, however, a bolus dose ratio for loss of the eyelash reflex in the presence of an opioid. It does not establish an appropriate dosing ratio for anesthesia maintenance.

Studies using propofol effect-site concentrations have quantified reductions in propofol concentration requirements associated with remimazolam coadministration. Zhou et al. allocated 150 women undergoing hysteroscopy to five groups that received intravenous remimazolam at 0, 0.05, 0.1, 0.15, or 0.2 mg/kg, followed by a sufentanil bolus of 0.15 μg/kg and propofol target-controlled infusion (TCI) [5]. The propofol effect-site concentration required to suppress the response to cervical dilation in 50% of patients decreased from 3.67 μg/mL without remimazolam to 2.08 and 1.43 μg/mL with remimazolam 0.1 and 0.2 mg/kg, respectively. Recovery time did not differ significantly among the groups. These findings demonstrate a reduction in the propofol concentration required to suppress a specific stimulus, namely, cervical dilation after induction. They do not establish the concentration combinations required for prolonged anesthesia maintenance.

In 2026, Zhang et al. studied 150 patients receiving sedation for gastroscopy without opioids [6]. Remimazolam 0–0.2 mg/kg was administered over 60 s, followed by propofol TCI targeting effect-site concentrations calculated using the Marsh model. Successful sedation was defined by loss of the eyelash reflex, a Modified Observer's Assessment of Alertness/Sedation (MOAA/S) score of ≤ 2, a bispectral index (BIS) of 40–60, and the absence of movement or coughing that interfered with the procedure. The median effective concentration (EC_50_) for this composite endpoint decreased from 3.81 μg/mL without remimazolam to 2.69 and 1.47 μg/mL with remimazolam 0.1 and 0.2 mg/kg, respectively. With remimazolam 0.1 mg/kg, weight- and duration-normalized propofol consumption also decreased from 0.587 to 0.414 mg/kg/min. Time from drug discontinuation to eye opening on command did not differ significantly between the group without remimazolam (7.9 min) and the group receiving 0.1 mg/kg (9.4 min), but was significantly longer in the groups receiving 0.15 and 0.2 mg/kg (11.0 and 10.6 min, respectively).These findings show that the combination that most reduces propofol consumption may not be the most advantageous when recovery is also considered.

A reduction in the propofol EC_50_ after adding another hypnotic can result from either additivity or synergy. Isobolographic or response surface analysis is required to determine whether the observed effect exceeds that expected from additivity. The nature of the interaction may depend on the endpoint assessed, such as a processed electroencephalographic index, loss of response to verbal commands or stimulation, or respiratory depression. Moreover, an EC_50_ based on a binary response, as in the studies described above, is the concentration at which half the population meets the endpoint. It does not represent a concentration that provides an adequate effect in nearly all patients.

Pharmacokinetics must also be considered when translating a bolus dose ratio into a continuous infusion regimen. When both drugs are given as boluses, or one as a bolus and the other by TCI, differences in their distribution and elimination cause the concentration ratio to change over time. The population pharmacokinetic model developed by Masui et al. provides a basis for predicting remimazolam concentration profiles [7]. Clinical application of an interaction therefore requires consideration of both the dose ratio and the concentrations of the two drugs at the time the effect was assessed.

For anesthesia maintenance, Obara et al. reported a retrospective series of 21 patients who received remifentanil, with a target Patient State Index (PSI) of 25–50. The median remimazolam infusion rate was 0.30 mg/kg/h, and the median predicted propofol effect-site concentration was 1.00 μg/mL [8]. Flumazenil was administered to four patients (19.0%): two before extubation and two after extubation. Among the 19 patients who did not receive flumazenil before extubation, the median time to recovery of responsiveness after discontinuation of both hypnotics was 13 min. This small, uncontrolled study supports the feasibility of the combination but does not establish an optimal regimen or quantify the interaction.

Processed electroencephalographic indices also require careful interpretation during combined administration. In a crossover trial in healthy adults, BIS values were higher with remimazolam than with propofol, even when clinical sedation depth was matched using MOAA/S scores [9]. This finding indicates that the relationship between BIS and clinical sedation depth differs between remimazolam and propofol. During combined administration, this relationship may also vary depending on the relative contribution of each drug. Therefore, findings from single-agent administration may not be directly extrapolated to combination therapy. Achieving the same BIS value in single-hypnotic and combination groups does not necessarily imply equivalent hypnotic effects. Evaluations of concentration–effect relationships during combined administration should necessitate the inclusion of raw electroencephalographic waveforms and spectral information alongside BIS, to correlate these metrics with behavioral responses whenever feasible.

Reversal must also be treated as part of the dosing regimen. Flumazenil antagonizes benzodiazepine effects, but it does not eliminate remimazolam from the body or reverse the residual effects of propofol. In a simulation-based analysis, Masui suggested that residual remimazolam effects due to reduced clearance, together with a large single bolus of flumazenil, may contribute to the reappearance of effects after reversal [10]. Studies of combined administration should record and evaluate not only when each hypnotic is discontinued but also the timing, dose, and criteria for reversal and the clinical course thereafter.

The relationships among concentration requirements, endpoints, and recovery described above are also evident with other hypnotic combinations. In a study of healthy volunteers, Dutta et al. found that dexmedetomidine reduced the propofol concentrations required to achieve behavioral endpoints, with the extent of reduction differing by endpoint [11]. In a clinical trial using BIS-guided closed-loop delivery, dexmedetomidine also reduced propofol consumption but increased the incidence of bradycardia and hypotension requiring treatment and resulted in greater sedation during early postoperative recovery [12]. Reduced drug requirements alone do not establish clinical benefit.

The combination of propofol and ketamine also illustrates endpoint-dependent interactions. Hui et al. administered the drugs alone or in combination to 180 women. They assessed loss of response to verbal commands 2 min after administration and loss of response to a 5-s transcutaneous tetanic stimulus. Additive effects were reported for both endpoints [13]. Sakai et al. showed that, when ketamine was added, clinical hypnotic endpoints could be reached with less propofol, yet BIS and the 95% spectral edge frequency (SEF_95_) could be higher at those endpoints [14]. Changes in electroencephalographic indices do not necessarily reflect the hypnotic effects of a drug combination. Achieving the same numerical target with different drug combinations therefore does not establish that the resulting anesthetic states are equivalent. Furthermore, in a recent observational study in which ketamine was added during stable propofol–remifentanil anesthesia, peak BIS and SEF_95_ values did not coincide with the peak predicted ketamine effect-site concentration. Instead, these peaks occurred later, while the predicted concentration was declining [15]. Interpretation of electroencephalographic indices during combined administration must therefore account for both the drug combination and the time elapsed since administration.

Propofol and sevoflurane have also been studied in combination. Using response surface analysis, Schumacher et al. reported additive interactions for reductions in BIS and entropy values and for suppression of responses to verbal commands and physical stimulation [16]. In a study of 72 women undergoing laparoscopic gynecological surgery, Uchinami et al. found no significant increase in postoperative nausea and vomiting when 0.8% sevoflurane was added to propofol, compared with propofol alone [17]. Clinical evaluation of a combination must therefore specify not only the drugs but also their concentrations and outcome measures.

The next step in evaluating propofol and remimazolam in combination is a prospective study of multiple concentration pairs, with sufficient observations near the single-agent conditions to estimate the interaction. Each endpoint, including loss of responsiveness to verbal commands, response to noxious stimuli, and electroencephalographic indices, should be assessed separately. Concomitant opioid administration should be specified, and assessments should be performed when the predicted effect-site concentrations of both hypnotics are sufficiently stable. Studies relying solely on fixed bolus doses are insufficient to accurately characterize the concentration–effect relationship during anesthesia maintenance. The estimated interaction should then be evaluated in clinical regimens that account for drug discontinuation and reversal when needed. The clinical value of combined administration depends on identifying conditions that maintain adequate hypnosis and allow timely emergence while providing measurable benefits, such as a reduction in adverse effects.

## Data Availability

Not applicable.
